# Modified Talk Test: a Randomized Cross-over Trial Investigating the Comparative Utility of Two “Talk Tests” for Determining Aerobic Training Zones in Overweight and Obese Patients

**DOI:** 10.1186/s40798-021-00315-9

**Published:** 2021-04-01

**Authors:** Ignacio Orizola-Cáceres, Hugo Cerda-Kohler, Carlos Burgos-Jara, Roberto Meneses-Valdes, Rafael Gutierrez-Pino, Carlos Sepúlveda

**Affiliations:** 1 Unidad de Fisiología Integrativa, Laboratorio de Ciencias del Ejercicio, Clínica MEDS, Santiago, Chile; 2 Applied Sports Science Unit, High-Performance Center, National Institute of Sports, Santiago, Chile

**Keywords:** Exercise prescription, Intensity, Aerobic training zones, Talk test

## Abstract

**Background:**

To validate the traditional talk test (TTT) and an alternative talk test (ATT; using a visual analog scale) in overweight/obese (OW-OB) patients and to establish its accuracy in determining the aerobic training zones.

**Methods:**

We recruited 19 subjects aged 34.9 ± 6.7 years, diagnosed with overweight/obesity (BMI 31.8 ± 5.7). Every subject underwent incremental cycloergometric tests for maximal oxygen consumption, and TTT in a randomized order. At the end of each stage during the TTT, each subject read out loud a 40 words text and then had to identify the comfort to talk in two modalities: TTT which consisted in answering “Yes,” “I don’t know,” or “No” to the question *Was talking comfortable?*, or ATT through a 1 to 10 numeric perception scale (visual analog scale (VAS)). The magnitude of differences was interpreted in comparison to the smallest worthwhile change and was used to determine agreement.

**Results:**

There was an agreement between the power output at the VAS 2–3 of ATT and the power output at the ventilatory threshold 1 (VT1) (very likely equivalent; mean difference − 1.3 W, 90% confidence limit (CL) (− 8.2; 5.6), percent chances for higher/similar/lower values of 0.7/99.1/0.2%). Also, there was an agreement between the power output at the VAS 6–7 of ATT and the power output at the ventilatory threshold 2 (VT2) (very likely equivalent; mean difference 11.1 W, 90% CL (2.8; 19.2), percent chances for higher/similar/lower values of 0.0/97.6/2.4%).

**Conclusions:**

ATT is a tool to determine exercise intensity and to establish aerobic training zones for exercise prescription in OW-OB patients.

## Key Points

- Aerobic training zones delimited by ventilatory threshold 1 (VT1) and ventilatory threshold 2 (VT2) were established through the alternative talk test (ATT) in overweight and obese people.
- Visual analog scale (VAS) 2–3 and VAS 6–7 identified VT1 and VT2, respectively, in overweight and obese people.
- ATT is a simple tool that could be applied to large populations due to its low cost and easy application and could be used for exercise prescription in community health centers.

## Background

Worldwide, low levels of physical fitness are associated with an increased risk of all-cause and cardiovascular disease [1], and physical inactivity is responsible for a substantial economic burden [2]. Also, poor cardiorespiratory fitness is an independent risk factor for developing non-communicable diseases (NCDs) and cardiovascular disease [3, 4]. Noteworthily, regular physical activity reduces the all-cause mortality risk by ~ 14% and is one of the leading non-pharmacologic strategies for preventing and treating obesity [1]. However, exercise prescription is complicated, depending on duration, frequency, intensity, and exercise type [5]. Moreover, under the same standardized training prescription, there are evident individual variations in post-training adaptations [6, 7]. Possibly, these individual training responses variations are dose-dependent. Therefore, the control of the physical training load is essential to maximize the benefits associated with health-related exercise [8].

There are several ways of prescribing exercise intensity, and some of these guidelines rely on objective criteria, such as percentages of absolute values of the heart rate reserve (%HRR) or the maximal oxygen uptake (VO2max). However, there is a convincing body of evidence suggesting that the relative distribution of training intensity is regulated more effectively based on the individual physiological and metabolic response to training [9, 10]. Exercise intensity determined by the individual physiologic and metabolic response (e.g., ventilatory threshold 1 [VT1], ventilatory threshold 2 [VT2], or lactate threshold) induces homogeneous training response and adaptations to training programs [11]. Besides, it is beneficial to identify these thresholds since it allows establishing the three classical training zones: zone 1, intensity < VT1; zone 2, intensities between VT1 and VT2; and zone 3, intensity > VT2 [12], favoring the control of external and internal training load [13, 14]. Despite the usefulness of laboratory evaluations individualizing exercise prescriptions, access to these measurements at the healthcare level is limited due to the cost of its implementation [15]. Therefore, research needs to develop low-cost and easy-to-apply training load control methods to improve access to individualized training programs in a specific population. In several countries, community healthcare centers do not have the equipment, infrastructure, and qualified professionals to assess these physiological variables. Moreover, probably the control of exercise intensity during a session only is evaluated with HR, %HRR, or rating of perceived exertion (RPE). However, if these methods are not associated with physiological and metabolic variables, the effectiveness of physical exercise could be diminished.

Low-cost methods, such as the talk test (TT) and the RPE, have been demonstrated to be of value relative to both performance diagnostics and prescription. Within the last years, the TT has been suggested as a useful surrogate of gas exchange thresholds in a variety of populations [16]. The TT is an easy-to-apply and low-cost tool for intensity monitoring [17] and involves an individual reading a similar text during exercise and then being asked if he or she can still speak comfortably [16]. However, the traditional talk test (TTT) only has three options for the question “was talking comfortable?” and lacks quantitative psychometric properties, being questioned as a substitute for objective physiological measures for prescribing individual training exercise [18].

The main objective of the study was to validate the TTT and an alternative TT (ATT; using a visual analog scale) in overweight/obese (OW-OB) patients and to establish its accuracy in determining the aerobic training zones, previously described. We hypothesized that ATT is a valid tool to establish aerobic training zones in OW-OB patients.

## Methods

### Participant Characteristics

A total of 19 subjects with a nutritional diagnosis of OW-OB according to BMI ≥ 25 kg/m^2^, physically inactive according to the World Health Organization (WHO) classification [19], and no diagnosis of NCDs participate in the study. Before the evaluations, the subjects signed an informed consent approved by the Scientific Ethics Committee of the Universidad Finis Terrae (resolution no. 21/2017). All procedures were performed in compliance with the Helsinki Declaration principles for human experiments.

### Study Design

The participants visited the laboratory in five separate days nonconsecutive (each evaluation day was intercepted per 3 rest days to prevent unexpected side effects as delayed onset muscle soreness “DOMS”). On the first day, the subjects signed informed consent. Next days, the subjects arrived between 08:00 and 10:00 am and were evaluated in a randomized order for the following procedures: body composition, cardiorespiratory fitness test, TTT, and ATT.

### Body Composition

Fat, lean, and fat-free body mass were measured by double-energy X-ray absorptiometry (DEXA) using manufacturer-supplied algorithms (Total Body Analysis, version 3.6; Lunar, Madison, WI, USA). The general characterization of subjects is presented in Table 1.

**Table 1:** Participant’s characteristics

	Total (*n* = 19) ♂ = 13, ♀ = 6
Age (years)	34.9 ± 6.7
Height (cm)	170.4 ± 7.8
Weight (kg)	89.5 ± 15.8
BMI (kg m^−2^)	30.8 ± 5.0
Fat mass (%)	37.0 ± 9.1
Lean mass (%)	59.4 ± 8.8
Fat-free mass (%)	63.0 ± 9.0

Data are shown as mean ± SD. Symbols: ♂ men; ♀ women

*Abbreviation*: *BMI* body mass index

### Cardiorespiratory Fitness

After a 5-min warm-up at 50 W and a constant cadence of 55 ± 5 rpm, the participants performed a maximal incremental test on an electronic automatized cycle-ergometer (Cyclus2, Germany). An initial workload of 40 W was used, with increments of 15 W (women) and 20 W (men) every 1 min until exhaustion. The test was performed with a constant cadence of 55 ± 5 revolutions per minute (rpm). Gas exchange was recorded continuously with a portable breath-to-breath gas analyzer (Cortex Metalyzer 3B, Leipzig, Germany) and was calibrated according to the manufacturer’s instructions before each trial. Pulmonary ventilation (VE), oxygen uptake (VO2), expired carbon dioxide (VCO2), and respiratory exchange ratio (RER) were averaged over 10 s in the mixing chamber mode, with the highest 30 s value (i.e., three consecutive 10 s averages) used in the analysis. VO2max was determined according to previously established criteria [20]: (i) plateau in VO2 (i.e., increase < 150 ml min^−1^), (ii) RER > 1.1, and (iii) ≥ 90% of theoretical maximal heart rate. The VO2max was expressed both as absolute values (L min^−1^) and relative to body mass (ml kg^−1^ min^−1^). The power output at VO2max (pVO2max) was determined as the minimum workload at which VO2max was reached. Ventilatory threshold 1 (VT1) and ventilatory threshold 2 (VT2) were identified separately by three researchers according to the following criteria [21]: an increase in VE/VO2 and end-tidal PO2 (PETO2) without a concomitant increase in VE/VCO2 for VT1, and an increase in VE/VO2 and VE/VCO2 and a decrease in end-tidal PCO2 (PETCO2) for VT2. The cardiorespiratory fitness and ventilatory threshold are shown in Table 2.

**Table 2:** Cardiorespiratory fitness and ventilatory threshold

	VT1	VT2	VO_2_max
VO_2_max (ml kg^−1^ min^−1^)	14.5 ± 2.5	22.4 ± 5.4	28.2 ± 7.0
METs	4.2 ± 0.8	6.3 ± 1.2	8.2 ± 2.0
VO_2_max (L min^−1^)	1.3 ± 0.3	2.0 ± 0.5	2.6 ± 0.7
% VO_2_max	51.8	77.1	96.5
Power (watts)	65.3 ± 20.8	133 ± 36.0	190.8 ± 55.3
HR (beats min^−1^)	109 ± 12	139 ± 15.0	163.6 ± 14.5
HR (%)	65.7	82.0	98.7
Ventilation (L min^−1^)	34.2 ± 8.1	59.5 ± 16.0	103.8 ± 28.7

Data are shown as mean ± SD

*Abbreviations*: *VO*_*2*_*max* maximal oxygen consumption, *VT* ventilatory threshold, *HR* heart rate, *METs* metabolic equivalent

### Talk Test

After a 10-min warm-up, subjects performed an incremental test on an electronic automatized cycle-ergometer (Cyclus2, Germany). The protocol considered load (W) increments every 3 min, the time necessary to stabilize ventilation, primary variable for voice production [22, 23]. During the last 30 s of each stage, out loud reading of 40 words from the text “Lectura del Abuelo” was requested. Two methods evaluated the ability to converse during exercise: (i) traditional talk test (TTT) by answering “yes,” “no,” or “I do not know” to the question “was talking comfortable?” and (ii) alternative talk test (ATT) using a 1 to 10 visual analog scale (VAS) [24]. Both, text “Lectura del Abuelo” and VAS, are shown in Table 3.

**Table 3:** Text “Lectura del Abuelo” and visual scale analog

Tex “Lectura El Abuelo”	Visual Scale Analog	
**El Abuelo**	**¿Fue fácil o difícil hablar?**	
“Usted quiere saber sobre mi abuelo. Bueno, él tiene cerca de noventa y tres años de edad y aún piensa tan lúcidamente como siempre. Se viste solo, y se pone su vieja chaqueta negra que comúnmente, tiene varios botones negros”	1	Extremadamente fácil
	2 – 3	Muy fácil
	4 – 5	Levemente difícil
	6 – 7	Difícil
	8 – 9	Muy difícil
	10	Extremadamente difícil
**Grandfather**	**Was easy or hard to talk?**	
“You want to know about my grandfather? Well, he is about ninety-three years old and still thinks as lucidly as ever. He dresses alone, and puts on his old black jacket, which usually has several buttons missing”	1	Extremely easy
	2 – 3	Very easy
	4 – 5	Slightly difficult
	6 – 7	Hard
	8 – 9	Very hard
	10	Extremely hard

le>

### Statistical Analyses

Data in the text and figures are presented as mean ± SD and 90% confidence limit/interval (CL/CI). A 90% confidence interval (CI; 1–2*α*) is used instead of a 95% CI (1–*α*) because magnitude-based inferences (MBI) performed two one-sided tests (each with an *α* of 5%). All data were first log-transformed to reduce bias arising from non-uniformity error. The magnitude of differences was interpreted in comparison to the smallest worthwhile change (SWC) (Cohen’s *d* = 0.6) [21]. Cohen’s *d* for within-subjects designs is calculated using the average standard deviation of both repeated measures as a standardizer with a Hedges’ correction to minimize bias (Cohen’s *d*_adj_) [25]:

$$ \mathrm{Cohen}'\mathrm{s}\ d\ \mathrm{adjusted}=\frac{Mdiff}{\left( SD1+ SD2\right)/2} $$

This SWC of 0.6 was set as the equivalence region, representing about one stage of difference during the incremental test, and was used to determine agreement. The probability of any substantial difference or realistic equivalence relative to the predefined target values was interpreted using the following scale: < 0.5%, most unlikely; 0.5–5%, very unlikely; 5–25%, unlikely; 25–75%, possibly; 75–95%, likely; 95–99.5%, very likely; > 99.5%, most likely [26]. Effects were declared relevant if the outcome probability was likely (≥ 75%) (i.e., methods were considered in agreement and, therefore, interchangeable). Statistical analysis was performed with the “mbir” package of the R software [27]. Statistical significance was set at *p* < 0.05.

## Results

We recruited 34 volunteer participants, of which 6 subjects were excluded because they did not meet the criteria for entering the study, and 9 did not complete all testing procedures. The final analysis, therefore, included 19 patients who completed the evaluations of which 13 were men and 6 were women.

### Agreement Between Traditional Talk Test and Ventilatory Thresholds

**Fig. 1:**
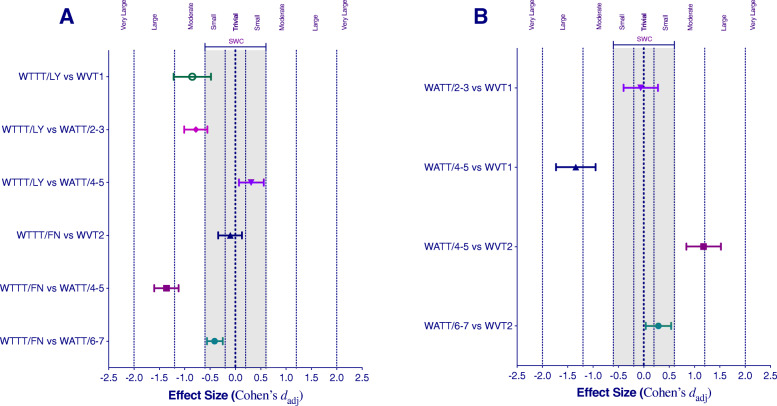
Mean difference and uncertainty for the difference (90% confidence interval) between **a** traditional talk test and ventilatory threshold and **b** alternative talk test and ventilatory thresholds. The unshaded area represents our statistical equivalence region. *Abbreviations*: *SWC*, smallest worthwhile change; *WTTT/LY*, watts of traditional talk test in the last stage where the answer was “yes”; *WVT1*, watts at ventilatory threshold 1; *WATT*, watts of alternative talk test; *WTTT/FN*, watts of traditional talk test in the first stage where the answer was “no”; *WVT2*, watts at ventilatory threshold 2; *W* watts

Results of the equivalence tests between TTT and ventilatory thresholds are presented in Fig. 1 and Table 4. Evidence for an agreement was observed between the power output at the “first no” (FN) and the power output at the ventilatory threshold 2 (most likely equivalent; mean difference − 2.9 W, 90% CL (− 10.9; 5.1)). There was no agreement between the power output at the “last yes” (LY) and the power output at the ventilatory threshold 1 (unlikely equivalent; mean difference − 22.4 W, 90% CL (− 1.3; − 13.3)). As represented in Fig. 1a, there was an agreement between the power output at the LY and the power output at the VAS 4–5 of ATT (very likely equivalent; mean difference 7.1 W, 90% CL (0.4; 13.7)).

**Table 4:** Analysis of agreement

Variable	Mean of differences	90% CI	Chances of true differences (%)	Effect size Cohen’s *d*_adj_ (90% CI)	Magnitude of effect	*p*-value
Traditional talk test: last stage where the answer was “yes”						
WTTT/LY vs WVT1	− 22.4	− 31.3/− 13.3	Unlikely equivalent (87.3/12.7/0.0)	− 0.85 (− 1.22/− 0.48)	Moderate	0.001
WTTT/LY vs WATT/2–3	− 21.1	− 27.5/− 14.5	Unlikely equivalent (90.3/9.7/0.0)	− 0.78 (− 1.01/− 0.55)	Moderate	0.000
WTTT/LY vs WATT/4–5	7.1	0.4/13.7	Very likely equivalent (0.0/97.1/2.9)	0.31 (0.07/0.56)	Trivial	0.039
Traditional talk test: first stage where the answer was “no”						
WTTT/FN vs WVT2	− 2.9	− 10.9/5.1	Most likely equivalent (0.1/99.9/0.0)	− 0.10 (− 0.34/0.13)	Trivial	0.455
WTTT/FN vs WATT/4–5	− 41.6	− 49.7/− 33.3	Most unlikely equivalent (100.0/0.0/0.0)	− 1.36 (− 1.60/− 1.12)	Large	0.000
WTTT/FN vs WATT/6–7	− 13.9	− 18.7/− 9.1	Very likely equivalent (1.9/98.1/0.0)	− 0.41 (− 0.56/− 0.25)	Trivial	0.000
Alternative talk test: visual analog scale						
WATT/2–3 vs WVT1	− 1.3	− 8.2/5.6	Very likely equivalent (0.7/99.1/0.2)	− 0.06 (− 0.40/0.28)	Trivial	0.763
WATT/4–5 vs WVT1	− 29.5	− 37.6/− 21.2	Most unlikely equivalent (99.8/0.2/0.0)	− 1.34 (− 1.73/− 0.95)	Large	0.000
WATT/4–5 vs WVT2	38.7	27.1/50.2	Most unlikely equivalent (0.0/0.4/99.6)	1.18 (0.84/1.52)	Moderate	0.000
WATT/6–7 vs WVT2	11.1	2.8/19.2	Very likely equivalent (0.0/97.6/2.4)	0.29 (0.04/0.54)	Trivial	0.058

*Abbreviations*: *SWC* smallest worthwhile change, *WTTT/LY* watts of the traditional talk test in the last stage where the answer was “yes”, *WVT1* watts at ventilatory threshold 1, *WATT* watts of the alternative talk test, *WTTT/FN* watts of the traditional talk test in the first stage where the answer was “no”, *WVT2* watts at ventilatory threshold 2, *W* watts

### Agreement Between Alternative Talk Test and Ventilatory Thresholds

Figure 1b shows an agreement between the power output at the VAS 2–3 of ATT and the power output at the ventilatory threshold 1 (very likely equivalent; mean difference − 1.3 W, 90% CL (− 8.2; 5.6)). There was no agreement between the power output at the VAS 4–5 of ATT and the power output at the ventilatory threshold 1 (most unlikely equivalent; mean difference − 29.5 W, 90% CL (− 37.6; − 21.2)). Results showed no agreement between the power output at the VAS 4–5 of ATT and the power output at the ventilatory threshold 2 (most unlikely equivalent; mean difference 38.7 W, 90% CL (27.1; 50.2)). As represented in Fig. 1b, there was an agreement between the power output at the VAS 6–7 of ATT and the power output at the ventilatory threshold 2 (very likely equivalent; mean difference 11.1 W, 90% CL (2.8; 19.2)). There was an agreement between the power output at the VAS 6–7 of ATT and the power output at the “first no” of TTT (very likely equivalent; mean difference − 13.9 W, 90% CL (− 18.7; − 9.1)).

## Discussion

The regulation and control of exercise intensity are some of the most challenging parts of exercise prescription. There are several ways of prescribing exercise intensity, and some of these recommendations are based on objective criteria, such as percentages of absolute values of the %HRR or VO2max. However, recent investigations propose a more individualized exercise prescription to personalize a training regime based on individual metabolic responses and, therefore, enhance the potential benefits of regular physical activity [11]. Therefore, the goal of this investigation was to prove the usefulness of the TTT and/or ATT as a low-cost tool to determine exercise intensity and establish aerobic training zones for exercise prescription in OW-OB patients.

Our main finding shows that the three aerobic training zones delimited by VT1 and VT2 could be established through the TT, primarily through the ATT. Regarding the TTT, previous studies have shown an association between VT1 and the last stage of the TT where talking was comfortable in healthy subjects [16, 28, 29] and between VT2 with TT stages where comfort to talk is lost in patients with heart diseases [30, 31]. However, these previous studies used the VO2 values to compare intensities between the TTT and ventilatory thresholds. This methodology does not allow obtaining external load values (e.g., watts) that can be used for prescribing and controlling aerobic training.

In our study, the TTT failed to determine the transition from zone 1 to zone 2 because we found no agreement between the power output of the different answers related to the TTT and the power output at VT1. The transition threshold between zone 2 and zone 3 could be established with the power output at the first stage where the answer was “no,” which was most likely equivalent to the power output at VT2. This lack of consistency with previous results could be related to the statistical analysis. The previous studies used correlation analysis (e.g., Pearson correlation) [16, 28], which focuses on the association of changes in two outcomes that often measure quite different constructs [32]. Our study used agreement analysis, which measures the degree of concordance in the results between two or more assessments of the variable of interest and assumes that the variables measure the same construct [32], being agreement analysis better to assess if methods are interchangeable [33]. To our knowledge, there is only one study in well-trained cyclists that measure agreement between workload at VTs and TT. Rodriguez et al. found agreement between the power output at the first stage where the answer was “I do not know” and the power output associated with VT1 [34]. Also, they found an agreement between the power output at the first stage where the answer was “no” and the power output associated with VT2, results that partially disagree with our recent findings in OW-OB patients.

Regarding the ATT, the absence of psychometric properties of the TTT may induce an under- or overestimation of the degree of talking comfort during physical exercise in physically inactive persons. The ATT would allow identifying the “difficult to talk” with numeric magnitudes by giving a quantitative variable to the TT [18, 35, 36]. Speech production during exercise is associated with changes in physiological variables related to exercise, being the consequence of the need to adapt the breathing pattern compatible with speech production. Accordingly, Rotstein et al. found a significant association between VO2, HR, and VE responses and the ratings of perceived speech production difficulty. Our results agree with these previous findings, showing that the intensity (power output) associated with VT1 is very likely equivalent to the last stage where talking was “very easy” (VAS 2–3), allowing to determine the threshold to zone 1. The power output at the first stage where talking was “hard” (VAS 6–7) was very likely equivalent to the power output at VT2 (Table 4), which allows determining the threshold to zone 3. The power output where talking was “somewhat hard” (VAS 4–5) was most likely higher than the power output at VT1 and most likely lower than the power output at VT2, thus representing the intensity related to zone 2 (Fig. 1b). Taken together, these results showed that the ATT could be used to determine exercise intensity and establish aerobic training zones for exercise prescription in OW-OB patients. The main limitation of clinical context is to have a low-cost tool to prescribe physical exercise. To solve this problem, ATT is a simple tool that could be applied to large populations due to its low cost and easy application. However, further research is needed to determine the effect of endurance training controlled with ATT on obese people.

Some limitations exist in this study as the low number of subjects recruited. Several studies have similar limitations [16, 34, 37]. Possibly the specific characteristics of them (e.g., participants with OW/OB) reduced the adherence of the participants. Another important issue to resolve for future studies is replicating the study on a treadmill with TTT and ATT in OW/OB patients with and without comorbidities. Interestingly, these tools could help healthcare professionals promote the massive practice of physical exercise in a population with the absence of technology to control the training program load. Finally, low-cost tools would help to improve the capacity of healthcare professionals to control exercise intensity, improving the health benefits of exercise and physical activity.

## Conclusions

ATT is a low-cost and easy-to-apply tool to determine exercise intensity and to establish aerobic training zones for exercise prescription in OW-OB patients. The TTT could under- or overestimate the physical effort in patients diagnosed with OW-OB, specifically at the training zone 1.

## Data Availability

Please contact the author for data requests.

## Abbreviations

%HRR: Percentages of absolute values of the heart rate reserve
ATT: Alternative talk test
BMI: Body mass index
CI: Confidence interval
CL: Confidence limit
FN: First no
HR: Heart rate
LY: Last yes
METs: Metabolic equivalent
NCDs: Non-communicable diseases
OB: Obese
OW: Overweight
PETCO2: End-tidal PCO2
PETO2: End-tidal PO2
pVO2max: Power output at VO2max
RER: Respiratory exchange ratio
RPE: Rating of perceived exertion
SWC: Smallest worthwhile change
TT: Talk test
TTT: Traditional talk test
VAS: Visual analog scale
VCO2: Expired carbon dioxide
VE: Pulmonary ventilation
VO2: Oxygen uptake
VO2max: Maximal oxygen uptake
VT1: Ventilatory threshold 1
VT2: Ventilatory threshold 2
WATT: Watts of the alternative talk test
W: Watts
WTTT/LY: Watts of the traditional talk test in the last stage where the answer was “yes”
WVT1: Watts at ventilatory threshold 1
WTTT/FN: Watts of the traditional talk test in the first stage where the answer was “no”
WVT2: Watts at ventilatory threshold 2
